# Development of a rapid field testing method for metals in horizontal directional drilling residuals with XRF sensor

**DOI:** 10.1038/s41598-021-83584-4

**Published:** 2021-02-16

**Authors:** Hailin Zhang, João Antonangelo, Chad Penn

**Affiliations:** 1 Department of Plant and Soil Sciences, Oklahoma State University, Stillwater, OK 74078 USA; 2 U.S. Department of Agriculture, National Soil Erosion Research, West Lafayette, IN 479072077 USA

**Keywords:** Environmental sciences, Environmental impact, Fluorescence spectroscopy, Sustainability, Characterization and analytical techniques

## Abstract

Portable X-ray fluorescence (pXRF) spectrometer allows fast in-situ elemental determination without wet digestion for soils or geological materials, but the use of XRF on wet materials is not well documented. Our objective was to develop a rapid field method using pXRF to measure metals in the residues from horizontal directional drilling (HDD) operations so that proper disposal decisions can be made in-situ. To establish the procedure, we spiked soil samples with 4 concentrations of Cr, Ni, Cu, Zn, As, Cd, and Pb up to 1000 mg kg^−1^, and then the metal concentrations were determined by wet chemical method after drying and acid digestion (standard method), and by pXRF, also at laboratory conditions, after drying and at two different moisture conditions. The measurements by pXRF and standard method after drying and after removal of excess water (AREW) were highly correlated with slopes ranging from 0.83 ± 0.01 to 1.08 ± 0.01 (*P* < 0.001) for all metals. The relationship was better AREW than the saturated paste without removal of excess water and the moisture content affected only the accuracy of As, Cd, and Pb. The procedure established was successfully used for HDD residues collected from 26 states of US with moisture content ranging from 14 to 83% AREW. The pXRF was proven to be a reliable tool for fast detection of common metals in dried soils and HDD residues, and samples containing < 30% moisture content without needing to correct for moisture. If the moisture is > 30%, excess water in samples need to be removed with a commercially available filter press to achieve high accuracy. The developed procedures reduce time of metal detection from days to about an hour which allows drilling operators to make quick decisions on soil or HDD disposal.

## Introduction

Horizontal directional drilling (HDD) and Hydro-Vacuum (HVac) “potholing” are two common pieces of equipment that are commonly used for the installation of underground pipe and cable utilities that are critical to life and industry in a modern society. The HDD is a trenchless technology^1^ with increasing worldwide demand^2^. According to the “20th Annual Directional Drilling Survey”, HDD will account for 50% of the pipeline trenchless installation market by 2023^3^. Hydro vacuum (HVac) is used to identify underground utility and pipes before any lateral ground disturbance take place. Spent HDD residues are an industrial by-product resulting from the drilling, which utilizes drilling fluid. The HDD residue contains both subsoil and some of the original drilling fluid constituents; water, bentonite (montmorillonite), and/or polymers^4,5^ as well as surfactants and soda ash when needed. These additives may or may not be utilized, depending on the type of formation that is being drilled through. The HVac residues are simply water and soil, which is vacuumed into a tank before disposal.

Excessive metal concentrations in HDD and HVac residues would only occur if the equipment is utilized in an area previously contaminated from historical industrial activity. However, although it will be rare for HDD residues to contain problematic concentrations of metals, it is impossible to definitively know the level of metals contained in the residues for timely disposal decisions. Many states do not allow disposal of drilling residues at the job site which requires expensive offsite disposal, such as landfill and designated sites. Such lack of knowledge might result in applying excessive levels of metals to a field if it is allowed. Therefore, the economic disposal of HDD residues is a growing concern due to negative perceptions by both environmental regulators and the public. Thus, alternative disposal methods, such as land application or as a compost amendment, must become viable and acceptable if proved that the material is not contaminated, which can be verified once the material is tested. However, routine laboratory testing is unpractical and time-consuming^6–10^ for on-site decisions.

A faster and non-destructive method of measuring metals in HDD residues in the field may be achieved with sensor technology by using a portable X-ray fluorescence analyzer (pXRF). This device would eliminate the need to digest samples from the HDD residues because metal concentrations can be measured directly on-site in the solid phase without tedious sample extraction in the laboratory^11–15^. It can detect many elements simultaneously on as-is samples. Some portable sensors employing x-ray fluorescence (XRF) spectrometry have been successfully used in mining and contaminated soils in-situ. The XRF technique has been adopted for solid waste analysis (SW-846 Test Method 6200: Field Portable X-ray Fluorescence), and soil and sediments (EPA Method 6200: Field Portable X-ray Fluorescence Spectrometry for the Determination of Elemental Concentrations in Soil and Sediment)^16,17^.

Since current procedures for using XRF are established for dry samples there is a need to make it useful for wet and even liquid samples because HDD residues are normally occurring as slurries with a wide range of moisture content, from 28 to 99%^18^. Thus, a procedure needs to be developed and tested for field measurements for metals in HDD and HVac residues by an XRF Spectrometer. Although some applications of pXRF are based on the ability to provide immediate measurements assuming no need for drying^19^, it has been demonstrated that water contents above 20% in soil or sediment could be a major source of error on absolute results^16,20^. However, the type of error and its relationship with water contents are not well documented. Some studies have shown that the accuracy of pXRF analysis is affected by soil moisture, organic matter content, particle size distribution and texture^7,12,21–23^. Currently, no convenient method is available to measure the onsite moisture level in HDD residues, thus little is known about potential effects of moisture contents on the accuracy of metal measurements.

For this study, we propose to evaluate a commercially available sensor and perform sample preparation processes with the goal of developing a quick and sufficiently accurate test method for determining metals in soils and HDD residual samples. Our objectives were (i) to establish a quick on-site metal determination method using pXRF at the appropriate moisture content; and (ii) to identify the accuracy and reproducibility of pXRF technique for HDD residual samples. The technique developed will allow drillers to quickly obtain critical information about the metal concentrations of their HDD so that disposal decisions can be made onsite.

## Methods

### Method establishment

To establish the relationship between pXRF measurement and standard wet chemical analysis (acid digestion and inductive coupled plasma atomic emission spectrometer, ICP-AES) for selected elements, regular soil samples were spiked with 7 elements at four concentrations up to 1000 mg kg^−1^. The soil samples used were collected from Perkins, Lake Carl Blackwell (LCB), Stillwater, and Coyle in Central Oklahoma (OK), US. All soils were sampled with an auger from the 0–15 cm soil layer, oven dried at 65 °C and ground to pass through a 2-mm sieve before metal spiking. Using methods described by Zhang and Henderson^24^, the soil pH was determined in deionized water with a 1:1 soil-to-water ratio and the organic matter (OM) by dry combustion using a LECO Truspec carbon and nitrogen analyzer (St. Joseph, MI). Soils initially possessed pH values of 5.6 ± 0, 6.1 ± 0, 5.7 ± 0.1, and 7 ± 0.2, and OM of 9 ± 0.6, 12.1 ± 0.5, 10.5 ± 0.2, and 29.1 ± 1.2 g kg^−1^, respectively, for soils from Perkins, LCB, Stillwater, and Coyle. Soil particle size distribution^25^ for all soils are detailed in Table 1. The first three soils (n = 12, 4 treatments × 3 replications) were used for method establishment.

**Table 1:** Particle size distribution, and moisture content in saturated paste and after removal of excess water from the saturated paste of the four soils used.

Soil	Particle size			Spiked metal concentrations (mg kg^−1^)	Moisture content	
	Sand (%)	Silt (%)	Clay (%)		Saturated paste (%)	After removal of excess water (%)
Perkins	73.7 (± 0.75)	18.4 (± 0.75)	8.4 (± 0.75)	0	15.4	11.3
				100	18.4	9.3
				250	19.5	7.5
				1000	17.1	11.3
LCB	35.9 (± 0.75)	46.7 (± 0.69)	17.5 (± 0.00)	0	25.3	18.1
				100	23.9	17.2
				250	26.7	17.1
				1000	24.5	19.3
Stillwater	55.9 (± 0.75)	29.2 (± 0.69)	15 (± 0.00)	0	21.7	13.4
				100	22.5	12.4
				250	22.3	12.9
				1000	22.0	12.9
Coyle	34.6 (± 0.69)	36.3 (± 1.3)	29.2 (± 1.4)	0	31.9	25.2
				100	30.6	23.9
				250	30.1	22.8
				1000	30.1	23.7

The soils were spiked with multiple metals for method development and validation. Moisture content is based on wet weight. ± is followed by the standard deviation of triplicates (n = 3).

The metals Cr, Ni, Cu, Zn, As, Cd, and Pb were added to 200 g of soils at approximately 0, 100, 250 and 1000 mg kg^−1^ w/w by using the reagents Cr(NO_3_)_3_.9H_2_O, Ni(NO_3_)_2_.6H_2_O, Cu(NO_3_)_2_.3H_2_O, Zn(NO_3_)_2_.6H_2_O, As_2_O_3_, Cd(NO_3_)_2_.4H_2_O, and Pb(NO_3_)_2_ and were well mixed in a 500 mL plastic container before saturation with water. The purpose of making the saturated paste was to enhance metal and soil interaction and simulate wet HDD resides to be analyzed by pXRF using the established method.

**Figure 1:**
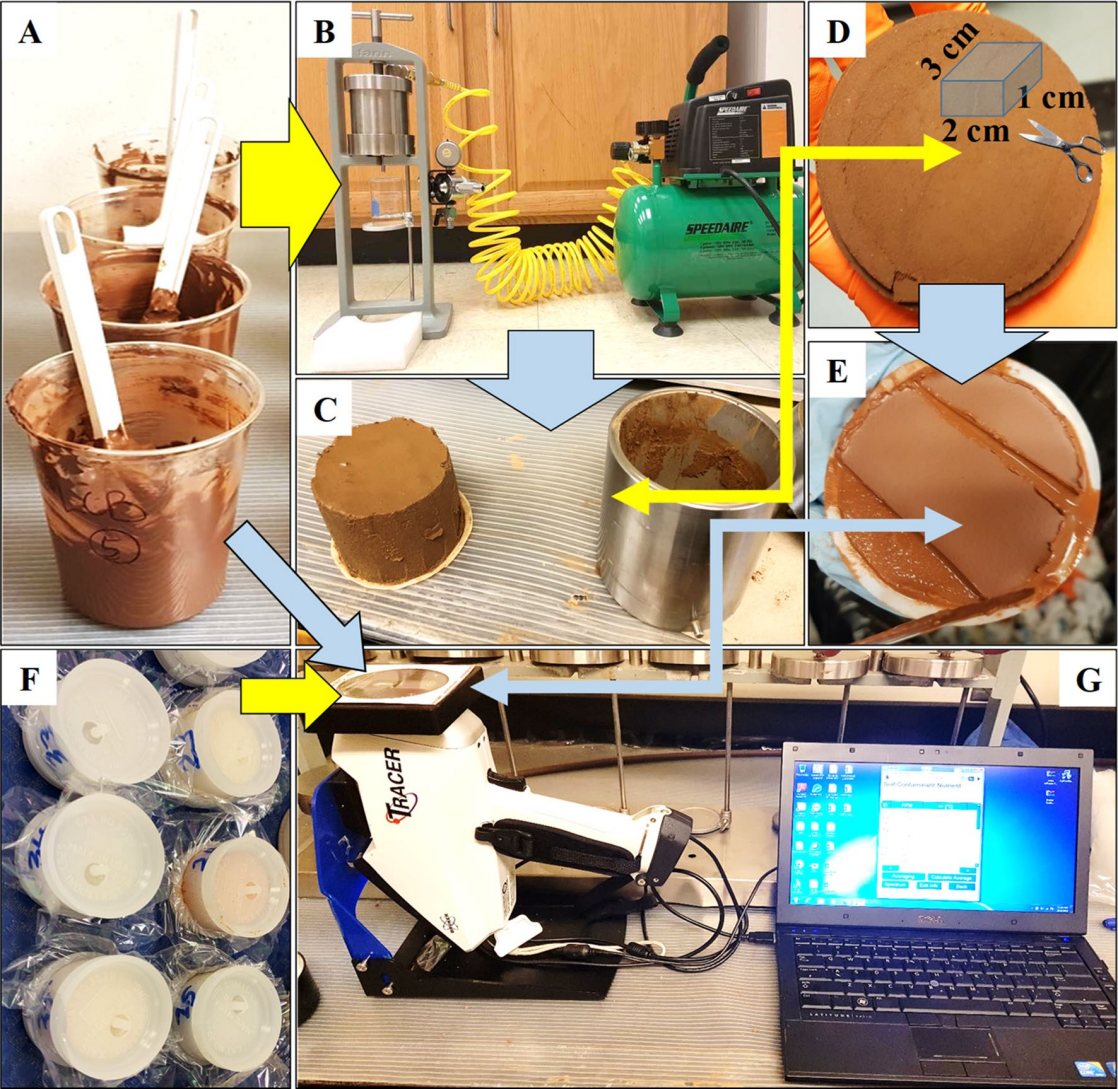
Flow diagram of metal detection in horizontal directional drilling (HDD) mud using a potable x-ray fluorescence spectrometer (pXRF). Saturated paste (**A**) of the soils or HDD residues is transferred into a metal cylinder mounted on a filter press and the excess water is removed by pressure using compressed air (**B**); then the moist sample is taken out of the cylinder (**C**) and properly cut (**D**) to fit in the sample holder and placed on the sensing window of the pXRF unit. The wet sample at the saturated paste (A) or after the removal of the excess water (**E**) and the same samples oven-dried at 105 °C and ground (**F**) were subjected to pXRF measurements (**G**).

To make the saturated paste, deionized water (D.I.) was slowly added while simultaneously stirred with a spatula until saturation point was achieved (Fig. 1). At this point, the soil pastes glistened as it reflected light but contained no excess water on the surface^26^. Samples were mixed after 1-h rest and then were covered and rested for 4 h to ensure equilibrium. The paste was scanned by a pXRF directly and also after removing excess water (AREW) with a filter press (Speedaire 0.33 HP, 115VAC, 2 gal. Portable Electric Oil-Free Air Compressor, 100 PSI) by pressurized air as shown in Fig. 1. Samples were placed in a metal cylinder mounted on a filter press and dehydration was performed by pressure using compressed air. The process to remove excess water required several minutes to an hour depending on the initial moisture and clay content. When necessary, the pressure was gradually increased to allow a progressive release of water. The filtrates were stored in a refrigerator prior to analysis. Moisture contents of saturated pastes and AREW for all soil samples are also shown in Table 1. Moisture content calculation was based on wet weight (or mass of wet soil/HDD residue), as shown in Eq. (1), because most of the actual HDD samples (for which this method is developed) contained more water than solids and it would result in > 100% moisture if the calculation was based on dry mass (or mass of dry soil/HDD residue). Therefore, “Wet weight basis” refers to results without correcting the soil moisture based on wet mass.

1 $$ {\text{Moisture }}\left( \% \right) \, = \left( {mass \,of \,water/mass\, of\, wet \,soil} \right) \times 100 $$

Subsamples of AREW soils were also oven-dried at 105 °C overnight, and ground for pXRF measurements (Fig. 1) and by acid digestion followed by metal analysis via ICP-AES for Cr, Ni, Cu, Zn, As, Cd, and Pb. The digestion was performed using nitric acid (EPA3050B), in which 0.5 g of each sample was predigested for 1 h with 10 mL of trace metal grade HNO_3_ in the HotBlockTM Environmental Express block digester. Digests were then heated to 115 °C for 2 h and diluted with deionized water to 50 mL. The AREW filtrates from the first three soils (Perkins, Lake Carl Blackwell (LCB), and Stillwater) were directly measured by ICP-AES to capture metals lost during the AREW process and to verify the recovery of metals spiked.

### Impact of moisture conditions on pXRF measurements

A second experiment was carried out by adding another soil (n = 16, 4 treatments × 4 replications), Coyle, with a greater clay content (Table 1) to verify the relationship between pXRF measurements at different moisture conditions: saturated paste (SP), after removal of excess water (AREW) from the saturated paste, and dried at 105 °C overnight and ground. All four soil samples were spiked with the same concentration of the metals as previously described. The Coyle soil with a greater clay content was added to cover the moisture content range present in soils and other geological materials studied elsewhere in the literature^17,19,27–30^ because HDD residues have a wide range of moisture content. Thus, there was a need to widen the range of moisture content to enhance the reliability of our study.

### Evaluation of pXRF methodology on HDD residues

Packages containing 1 L sample bottles, instructions on how to obtain a representative sample, and a written survey were sent to contractors. Fifty-nine samples were received from 26 US states. The moisture contents of HDD residues ranged from 28 to 96% with mean and median values of 65%, and 66%, respectively, which suggests a normal distribution of the solid/moisture contents. The broad range of moisture content was due to the variability of the original drilling fluid used and the various formations or subsoils being bored through. Excess water must be removed in order to directly use XRF for metal determination. Electrical conductivity (EC) and pH of HDD samples were tested using standard EC and pH meters. The EC of the residuals ranged from 118 to 3950 µS cm^−1^ with mean and median values of 1198 and 1124 µS cm^−1^, respectively, while pH values were similar to that of a typical subsoil ranging from 4.7 to 9.9 with a mean of 7.5 and a median of 7.6.

The same procedures of excess water removal were performed on HDD residual samples with a filter press (Fig. 1). The AEWR samples were treated the same as the previously described soil samples for analysis of Cr, Ni, Cu, As, Zn, and Ba by pXRF and digestion.

### X-ray fluorescence instrument and measurements

All pXRF measurements were performed using a TRACER 5i Portable/Handheld XRF Spectrometer (Bruker, Kennewick, WA, USA). The energy dispersive X-ray fluorescence (EDXRF) technology uses an X-ray tube as their excitation source. When energized, the instruments generate low-energy X-rays. A Cu 200 µm:Ti 25 µm:Al 300 µm filter, inserted into the filter holder, is attached to the collimator. The collimator, which determines the size of the location on the sample to be tested, has a diameter of 8 mm, which is ideal for avoiding reduction of the focal point size and, therefore, the analyzed area.

A Rhodium (Rh) thin window X-ray tube was the excitation source (X-ray generator 6–50 kV with 4.5–195 uA, maximum 4 W output). The detector window was an 8 μm Beryllium. The TRACER 5i pXRF detects from Na to U and analyzes from Mg to U. All analyses were performed using the Soil Nutrient and Metal calibration provided by the manufacturer. Readings were taken at 50 kV, 39 µA, with a Duplex 2205 sample over the examination window and rounded up to the nearest 5 µRem/hr value. Samples were analyzed for 60 s using 2 phase scans of 30 s each phase. For laboratory measurement, the pXRF gun was mounted upside down on a stand, and moist samples were cut to size about 2 × 3 × 1 cm and directly placed on the sensing window while dried samples were packed in the holder and then placed on the window (Fig. 1).

### Quality control

The analyses included laboratory check samples for quality assurance and quality control (QA/QC). Reagent blanks and reference samples were analyzed for every nine samples. If a check sample failed control limits, then the nine samples analyzed before and after the failure were reanalyzed. Certified biosolids-treated reference soil (CRM005–050, RTC Corp., Laramie, WY, USA) and Metal-rich reference sediment (SdAR-M2, International Association of Geoanalysts, Keyworth, Nottingham, UK) were included in the determination of the total elemental contents in the liquid and solid portions, where applicable, of soils and HDD residues by ICP and pXRF, respectively. Acceptable method blank concentrations of all elements analyzed were below the established instrumental Limits of Detection (LOD), and the LOD of metals were in the range of 0.1–0.2 mg L^−1^ as per ICP measurements. The LOD of the pXRF is shown in Table 2. Supplementary Table S1 shows the recovery of metals spiked, which was calculated using delta variation (∆%), where ∆% = ([metal content AREW_(ICP or pXRF)_ + metal content in the filtrates_(ICP)_] – [target initial concentration of spiked metals]) made to percentage.

**Table 2:** Summary of elemental K-edge absorption energies that were scanned under 50 keV pXRF analysis settings, the limit of detection (LOD) of metals for the pXRF used, mean metal concentrations found in typical Oklahoma soils^31^, and EPA (Environmental Protection Agency) limits of metals for biosolids.

Element	K-edge (keV)	LOD (pXRF)	Mean concentration (range) in OK Soils	EPA biosolids exceptional quality
		mg kg^−1^		
Cr	5.989	< 5	26.2 (4.3–69.7)	n/a
Ni	8.333	< 5	16.9 (2.4–57.3)	420
Cu	8.979	< 5	12.6 (1.9–35.7)	1500
Zn	9.659	< 5	55.2 (15.3–142)	2800
As	11.867	< 5	6.25 (0.75–33.6)	41
Cd	26.711	< 20	0.30 (0.13–0.8)	39
Pb	88.005	< 9	14.9 (2.6–31.7)	300
Ba	37.441	< 160	–	–

*pXRF* portable XRF.

*n/a* not applicable.

“–”: not available.

The check samples and certified materials validate the instrument's accuracy during measurements. As presented by the manufacturer, the pXRF does not need to be recalibrated, except when a component is changed or fails, then the calibration has to be redone. Therefore, the use of the check sample that accompanies the instrument and/or the certified standards are enough for geoscience applications given the strong stability of the instrument during field operations over time. It is recommended to run a check sample at the beginning and end of shift to ensure no more than 1 day passes before a problem can be observed. The reliability of calibration depends on the variability of the reference standards and resemblance to the sample matrix^32^. Hence, the accuracy of the instrument will strongly depend on the similarity between the chemical composition of the analyzed samples and factory calibration standards. Thus, no user calibration is required when adequate one-time factory calibration is used^33^. In our experiment, the proper check sample (SdAR-M2, International Association of Geoanalysts, Keyworth, Nottingham, UK) and/or certified material (CRM005–050, RTC Corp., Laramie, WY, USA) were used, as previously stated.

### Data analysis

For each treatment (spiked concentrations of metals), the standard deviation (SD) of replicates considered all soils with the same spiked concentration of metals. The SDs were plotted in column graphs as error bars to show differences between pXRF measurements under different moisture conditions. Simple linear regression models were adopted to evaluate the relationship between the different methods of measurements and were verified by the two-tailed test at α = 0.05. The root mean square error (RMSE), normalized RMSE (NRMSE), and d-index were calculated by Eqs. (2), (3), and (4), respectively, to validate the models adopted. Before the regression analysis, data were ensured to be normally distributed by the Shapiro–Wilk test.

2 $$ {\text{Root}}\,{\text{ mean }}\,{\text{square}}\,{\text{ error}}{:}\, { }RMSE = \sqrt[{}]{{\frac{{\mathop \sum \nolimits_{i = 1}^{n} \left( {P_{i} - O_{i} } \right)^{2} }}{n}}} $$

where *Pi* is the *i*th predicted value, *Oi* is the *i*th observed value, and *n* is the sample size.

3 $$ {\text{Normalized}}\,{\text{ root }}\,{\text{mean}}\,{\text{ square}}\,{\text{ error}}:\,{ }NRMSE = \frac{RMSE}{{\overline{O}}} $$

where *Ō* = is the mean of observed values. If the value of NRMSE is less than 10%, the degree of the fitness is considered excellent; if 10% ≤ NRMSE < 20%, the degree of the fitness is considered good; when 20% ≤ NRMSE < 30%, the degree of the fitness is considered common; and if the value NRMSE is larger than 30%, the degree of the fitness is considered poor^34,35^.

4 $$ {\text{d-index}} = 1 - \left[ {\frac{{\mathop \sum \nolimits_{i = 1}^{n} \left( {P_{i} - O_{i} } \right)^{2} }}{{\mathop \sum \nolimits_{i = 1}^{n} \left( {\left| {P_{i} ^{\prime}} \right| + \left| {O_{i} ^{\prime}} \right|} \right)^{2} }}} \right] 0 \le d \le 1 $$

where *n* is the sample size, *Pi* is the proposed method value, *Oi* is the standard method value, *Ō* = the overall mean of the proposed method value. *Piʹ* is *Pi* – *Ō*, and *Oiʹ* is *Oi* – *Ō*, where the overall mean of the proposed method (*Ō*) is deducted from every single value of the standard method (*Oi*). The d-index varies between 0 and 1, with a value of 1 indicating perfect agreement between proposed (pXRF) and standard methods (digestion)^36^.

## Results

### The relationship between pXRF and standard method

**Figure 2:**
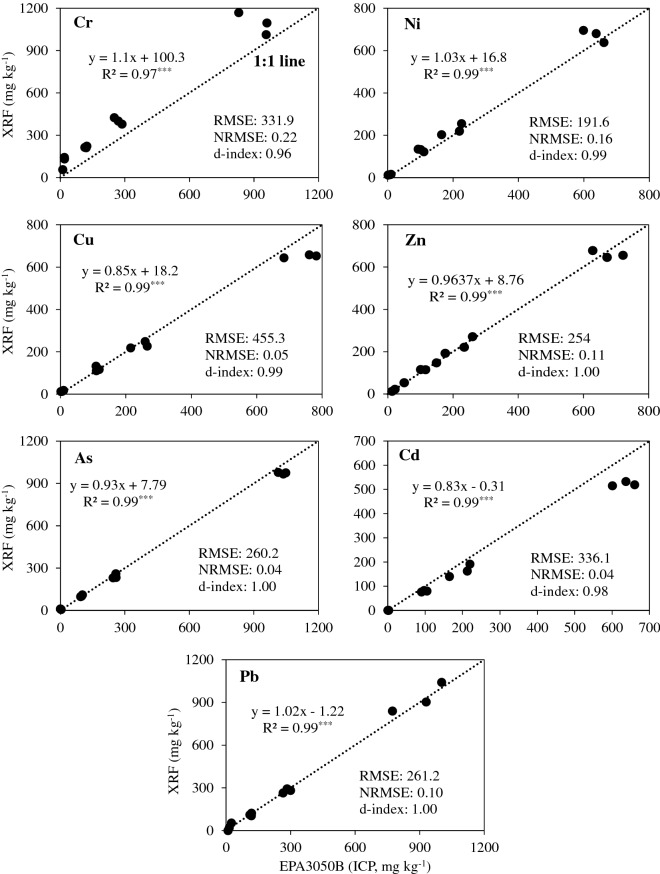
Linear regressions and statistical parameters between metal concentrations obtained with ICP (EPA Method 3050B) and direct measurement by a portable XRF after removing excess water from saturated paste with a pressurized press and oven drying at 105 °C (n = 12). ***: *P* < 0.001. Dashed lines are 1:1 lines.

The first 3 soils listed in Table 1 were spiked with metals at 4 different concentrations and allowed to equilibrate at saturation. Figure 2 shows the positive 1:1 relationship between standard (ICP) and proposed (pXRF) methods of measurements using oven-dried and ground samples AREW. All slopes were significant (*P* < 0.001) and close to 1 ranging from 0.83 ± 0.01 to 1.08 ± 0.01 for all metals. The intercept of those relationships ranged from 7.8 ± 4.4 to 100.3 ± 29.2. Intercepts greater than zero indicates that some Cr, Ni, Cu, Zn, and As are naturally occurring in the soil samples used. The normalized root mean square error (NRMSE) (Eqs. 2 and 3) ranging from 4 to 22% indicates a degree of fitness in the excellent to the common level^34,35^. Moreover, the d-index^36^ (Eq. 4) showing values between 0.96 and 1.00 indicates a perfect agreement between proposed and standard methods (Fig. 2). These results confirm the established pXRF measurements for the metals studied are accurate compared with the standard wet chemistry method. The direct pXRF measurements using moist samples AREW (without oven drying) were similar to those of dried samples (data not shown), suggesting that it is possible to estimate the concentrations of all metals from pXRF measurements on samples with moisture contents between 9.9 ± 1.8 and 17.9 ± 1% without oven drying.

### pXRF measurement of metals under different moisture conditions

Another soil with a greater clay content was added to the previous used 3 soil samples (4 soils total) to evaluate the relationships between pXRF measurements at the moisture conditions of the saturated paste (SP) before removal of excess water, and that AREW. The moisture content at saturation increased as the clay content increased in a linear relationship of: Water saturation (%) = [(0.62 × Clay content, in %) + 12.9] (R^2^ = 0.98, *P* < 0.001). The moisture content for the saturated pastes ranged from 17.6 ± 1.8 to 30.7 ± 0.9%, that of AREW were reduced to 9.9 ± 1.8 to 23.9 ± 1% (Table 1). As expected, soils containing a greater clay content retained more water during the process of removing excess water with pressure than those with lesser clay contents^37^. Water removal by pressure extraction was 28.6% for LCB and 22.1% for Coyle. Meanwhile, Perkins and Stillwater lost 44% and 41.7% of water, respectively.

**Figure 3:**
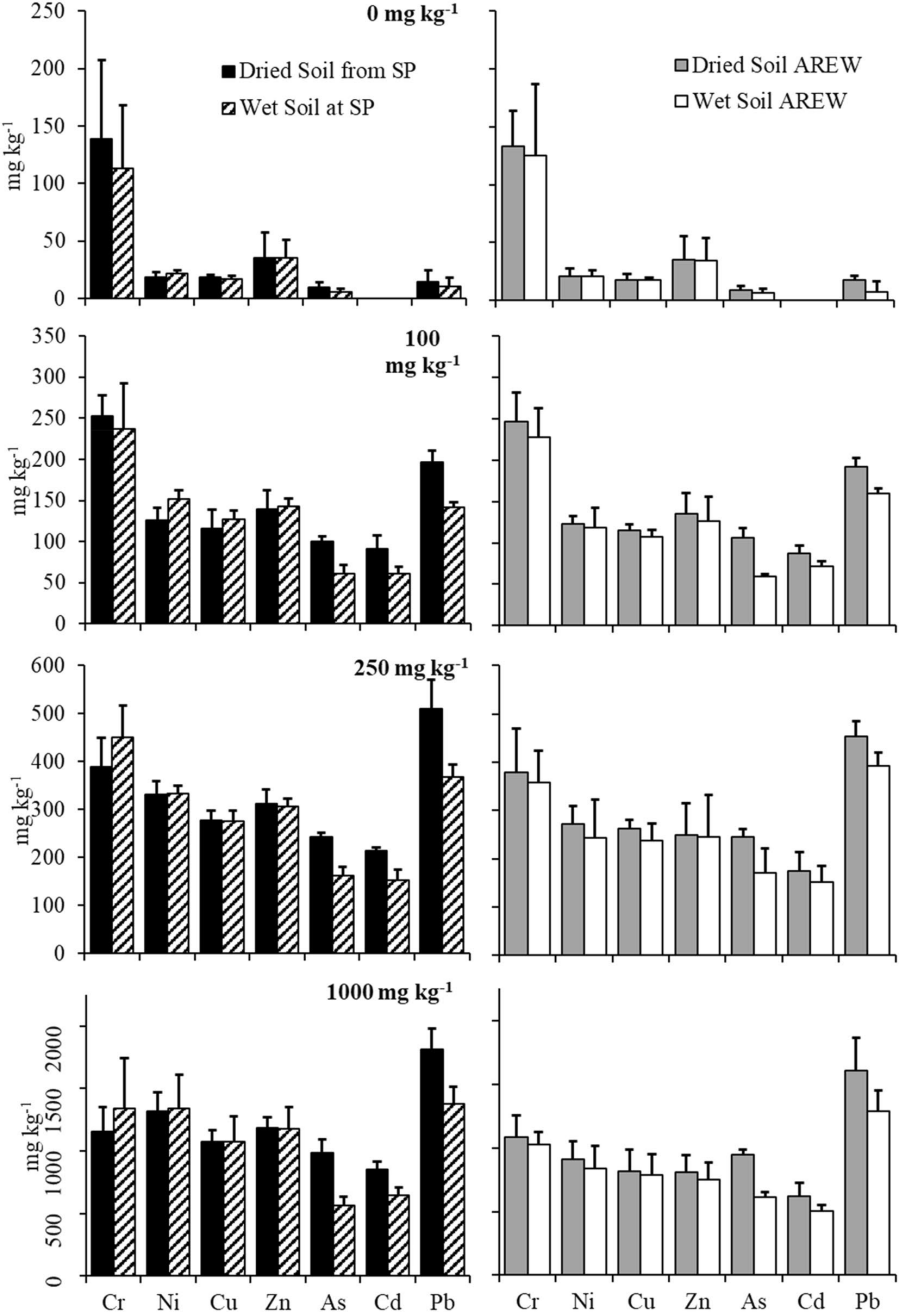
Metal concentrations determined by pXRF of oven-dried and wet soils at the saturated paste (SP) (left) moisture condition and after removal of excess water (AREW) from saturated paste (right). Bars are the standard deviation of the four soils (n = 4) with clay textures ranging from 8.4 ± 0.8 to 29.9 ± 1.4%. Treatments of metals ranged from 0 to 1000 mg kg^−1^ (except Pb which was higher than others), and the values for the 0 treatment are the natural background concentrations of the soils used.

Concentrations of metals measured with pXRF, obtained from saturated pastes and from samples AREW were compared to their corresponding dried samples (Fig. 3). The dilution effect attributed to wet samples is noticeable for As, Cd, and Pb in all situations, except for Cd in the control since it was below the limit of detection. The spiked Pb concentrations (0, 172, 428, and 1719 mg Pb kg^−1^) were different from others due to an error in calculation but the increased amount of Pb does not affect the comparison of the two methods evaluated.

The relationships between pXRF measurements of oven-dried (dry) samples (x-axis) with wet samples of both SP and AREW (as-is moisture content i.e. wet weight basis) and concentrations of wet samples after correcting for moisture presented (dry weight basis) (y-axis) are shown in Table 3.

**Table 3:** Statistical parameters of the linear regression between metal concentrations in soils oven-dried at 105 °C (x-axis) and soils under different moisture conditions by pXRF measurements (y-axis).

y-axis	Saturated paste (SP)					After removal of excess water (AREW)				
	Intercept	Slope	R^2^	NRMSE	d-index	Intercept	Slope	R^2^	NRMSE	d-index
**Cr (mg kg**^**−1**^**)**										
Wet weight basis	− 61.10	1.23	0.96***	0.20	0.97	8.84	0.92	0.97***	0.15	0.99
Dry weight basis	− 63.00	1.58	0.98***	0.15	0.90	4.94	1.13	0.95***	0.20	0.98
**Ni (mg kg**^**−1**^**)**										
Wet weight basis	7.86	1.01	0.97***	0.21	0.99	1.18	0.92	0.96***	0.22	0.99
Dry weight basis	18.90	1.30	0.98***	0.15	0.96	− 5.50	1.13	0.96***	0.22	0.98
**Cu (mg kg**^**−1**^**)**										
Wet weight basis	1.28	1.00	0.98***	0.19	0.99	− 3.92	0.96	0.98***	0.14	0.99
Dry weight basis	7.40	1.30	0.99***	0.11	0.97	− 14.16	1.19	0.98***	0.17	0.98
**Zn (mg kg**^**−1**^**)**										
Wet weight basis	− 0.72	0.99	0.99***	0.12	1.00	6.21	0.92	0.98***	0.15	0.99
Dry weight basis	5.70	1.29	0.99***	0.05	0.97	0.47	1.14	0.98***	0.16	0.99
**As (mg kg**^**−1**^**)**										
Wet weight basis	8.43	0.57	0.99***	0.11	0.88	1.14	0.65	0.98***	0.17	0.92
Dry weight basis	14.70	0.73	0.98***	0.15	0.96	− 0.68	0.78	0.99***	0.12	0.97
**Cd (mg kg**^**−1**^**)**										
Wet weight basis	− 5.70	0.76	0.99***	0.09	0.96	6.78	0.79	0.98***	0.15	0.97
Dry weight basis	− 4.90	0.98	0.99***	0.07	1.00	3.95	0.97	0.99***	0.09	1.00
**Pb (mg kg**^**−1**^**)**										
Wet weight basis	− 6.00	0.76	0.99***	0.14	0.96	11.39	0.79	0.99***	0.08	0.98
Dry weight basis	− 2.30	0.98	0.99***	0.13	1.00	0.37	0.98	0.99***	0.06	1.00

Samples were grouped as ‘saturated paste’ (SP), and ‘after removal of excess water’ (AREW), with moisture content ranging from 17.6 ± 1.8 to 30.7 ± 0.8% and 9.9 ± 1.8 to 23.9 ± 1% based on wet weight.

***Significant at *P* < 0.001.

Expressing metal concentrations on a dry weight basis improved the slope (closer to 1) for As, Cd, and Pb, but overestimated for Cr, Cu, Ni and Zn (slopes > 1) for the saturated paste samples (Table 3). However, using dry weight basis did not change the slope for any metals for the samples AREW suggesting correcting for moisture is not necessary if excess water in the samples is removed using the filter press. The linearity of the relationship between samples AREW and dried samples confirms it is reliable to determine metal concentrations on field-prepared samples and the relationship is better than that between wet (SP) and dried samples. The NRMSE for all metals was less than 22%, which denotes a common level of degree of fitness; and most NRMSE values were in the ranges of 10–20% and 0–10%, highlighting good and excellent levels of degree of fitness, respectively. In addition, d-indexes were overall close to a perfect agreement (d-index =  ~ 1.00) (Table 3).

### Using pXRF to measure metals in HDD residues

**Figure 4:**
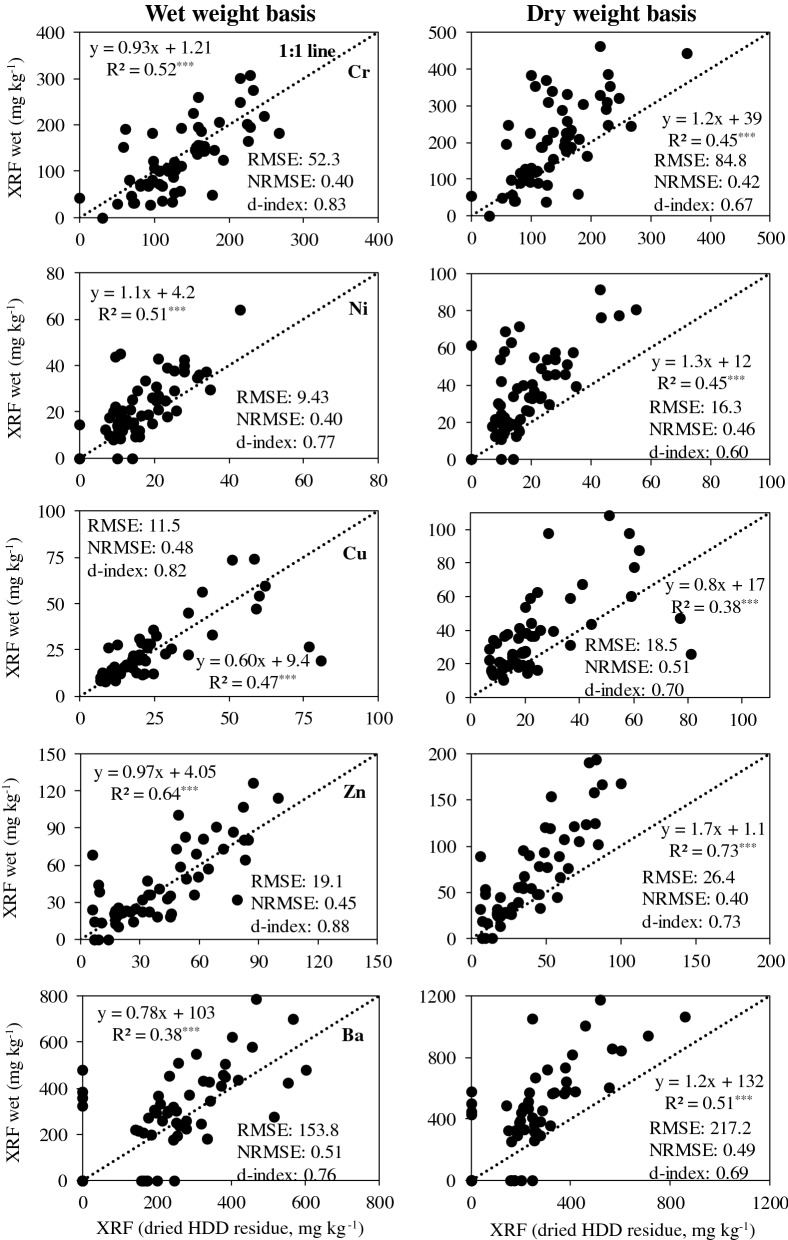
Linear regressions and statistical parameters between metal concentrations determined by pXRF on oven-dried and moist (wet) samples after removing excess water from 59 horizontal directional drilling (HDD) residues collected from 26 states of the US (a few outliers were removed under the normality assumption). ***: *P* < 0.001. **: *P* < 0.01.

The 59 actual HDD residues were subjected to pXRF scans using the procedure established by removing excess water with a compressed air press. Not all metals evaluated previously were detectable in the HDD residues but Cr, Ni, Cu, and Zn as well as barium (Ba) were detectable (Fig. 4). The maximum concentrations found for Cr, Ni, Cu, and Zn by pXRF in dried HDD samples were approximately 300, 50, 90, and 120 mg kg^−1^, respectively, which were within the ranges used for method development (0 to 1000 mg kg^−1^).

When evaluating the relationship for pXRF measurements between oven-dried (dry) and wet samples AREW (as-is moisture content), the NRMSE values ranged from low to very high, but d-indexes for Cr, Ni, Cu, Zn, and Ba were considered high at 0.83, 0.77, 0.82, 0.88, and 0.76, respectively (Fig. 4). Consequently, the slopes of linear regression models between dry samples and wet samples AREW were close to 1, except for Cu and Ba due to some outliers. Overall, these results are in agreement with the findings presented in the previous step where metals under consideration could be directly measured by pXRF in wet samples without the need for determining moisture content, even when moisture content varied from ~ 15 to 30%. The range of moisture content for AREW HDD residues was much wider than the soils used to establish the procedure, varying from 13.7 to 83.2%, which is probably due to a higher clay content in some samples and required longer time to remove excess water. Thus, it may be less accurate for some metals because the water content is higher than the desired level (< 30%). As previously mentioned, HDD residues contain bentonite clay, a 2:1 soil mineral with high water holding capacity. The clay could seal off the filter in the press used to remove excess water and extend the time needed to remove excess water. The relationships between oven-dried and wet HDD residues expressed on a dry weight basis are also shown in Fig. 4 and demonstrate that correcting moisture does not improve accuracy based on the R^2^, slopes, NRMSE, and d-index values.

## Discussion

The highly significant relationship between metals determined by pXRF and the standard acid digestion method by ICP (Fig. 2) suggests pXRF is a reliable tool for dried soil samples. However, the drying and grinding process is time consuming due to the high moisture contents of HDD and does not allow for making onsite decision for HDD disposal. Therefore, a procedure to determine metal concentrations by pXRF on samples with different moisture conditions is desirable for the directional drilling industry which generates large quantities of drilling mud.

Moisture content affected the accuracy of the As, Cd, and Pb measurements by pXRF, probably due to sample dilution since wet soil samples had lower values than those of oven-dried (dry) soil samples. It was identified in the significantly < 1.0 slopes observed for As, Cd, and Pb when comparing results from wet soil samples expressed on a wet weight basis with results of dried samples (Table 3). Especially for elements with low energy X-ray lines (< 5 keV), the measured concentration decreases as the water content increased. In the case of our experiment, moisture contents decreased from 17.6 ± 1.8 to 30.7 ± 0.9% for the SP to 9.9 ± 1.8 to 23.9 ± 1% for AREW and therefore accuracy improved by removing excess water. However, such effect may be counterbalanced by the reduced matrix absorption as a consequence of large matrix heterogeneity associated with soils since As, Cd, and Pb actually exhibit K-edge absorption energies > 5 keV (Table 2). Therefore, the bias introduced by moisture may also be dependent on the target element and matrix composition^19,38^.

Our data suggest that measurements on samples AREW produced by a filter press using pressurized air and possessing water contents of up to ~ 30% can provide reliable results of common metals in surface and subsurface soils. The mud should be permitted to be applied on nearby land if no metal is over any regulatory limit. Otherwise, it should be disposed according to local environmental regulations. Although the concentrations determined by pXRF are slightly different from those of the standard method, it is in the acceptable range^19^. Moreover, the slopes between samples tested on oven-dried and SP for As, Cd, and Pb were 0.57, 0.76, and 0.76, and improved to 0.73, 0.98, and 0.98 after correcting moisture (i.e. express as dry weight basis) in SP (Table 3). Similarly, such improvements were also observed for measurements conducted on samples AREW. Thus, moisture corrections can be used for pXRF measurements conducted on moist samples. Moisture determination can be achieved on site by heating a small amount of sample in a microwave oven for about 3 min^39^. Therefore, pXRF technique allows for on-site metal determinations with results obtained in about an hour^17^.

When a filter press is needed, the metals lost may need to be accounted for since it will depend significantly on the metal contents and soil/other geological materials' chemical and physical properties. In the case of our study, the first experiment of method establishment showed that metals were not lost significantly when the control treatments (no metal-spiked soils) were filtered, and they contained the background concentration of HM (Supplementary Table S1 and Fig. 3), which was similar or lower than those presented in the actual HDD residues. Therefore, metals in the filtrate of actual HDD samples can be neglected unless it is contaminated.

When evaluating the relationships between pXRF measurements using oven-dried and moist samples AREW without moisture correction for the HDD residues (Fig. 4), the findings were as good as those obtained with the spiked soils even with a wider moisture content in the HDD residues ranging from 13.7 to 83.2% AREW (based on wet weight). However, correcting the remaining moisture AREW did not improve the relationships significantly, which is probably a consequence of the different matrix composition of HDD residues when compared to soil samples. Therefore, onsite soil drying to obtain moisture is not necessary. Lemiere et al.^19^ also observed that the measurements of metal contents in wet samples of sediments were correlated with metal contents in dry samples even at moisture levels between 30 and 70%.

As a matter of fact, expression of metal concentrations based on wet weight is acceptable and more practical than dry weight for HDD residues, as the regression between dried samples and wet samples expressed on a dry weight basis only slightly changed the slope values for Cr, Ni, Cu, Zn, and Ba from 0.93, 1.1, 0.6, 0.97, and 0.78 to 1.2, 1.3, 0.8, 1.7, and 1.2 when wet samples were expressed on an as-is wet basis. These small changes in slope occurred without substantial changes in the NRMSE and d-index values (Fig. 4). Thus, correcting moisture made no differences for 2 elements but overestimated for the other 3 elements. This emphasizes that correcting for remaining moisture AREW from HDD samples may not be needed.

Although the limits of detection (LOD) of the pXRF are not as low as ICP for the metals tested they are far below the allowed maximum concentrations of biosolids for land application (Table 2). None of the HDD materials were considered to be problematic based on EPA standards for biosolids or superfund sites^18^. In fact, all of the HDD residues tested are considered “exceptional quality” according to the EPA 503 rule on biosolids (Table 2). Although some HDD samples were elevated in metal concentrations, they were comparable to the background levels in Oklahoma soils (Table 2). Onsite pXRF measurement should serve as an accurate assessment for total metal contents in soils and HDD samples. Therefore, allowing for a decision to be made quickly regarding disposal.

## Conclusion

The pXRF was proven to be a reliable tool for onsite detection of common metal concentrations in soils and HDD residues (mud) with approximately < 30% moisture content. If the moisture is higher than 30%, excess water in samples needs to be removed with a commercially available filter press to achieve high accuracy. Although metals are determined on moist samples, it is unnecessary to correct for the remaining moisture by measuring moisture content. The procedures developed reduces sample metal detection from days to about an hour for most samples which allows drilling operators to make immediate decisions on soil or HDD and HVac residue disposal.

## Supplementary Information

Supplementary Information

## Data Availability

Correspondence and requests for materials should be addressed to H.Z.

## References

[1] Dong S (2020). Experimental and performance analysis of reverse circulation reaming in horizontal directional drilling. Tunn. Undergr. Space Technol..

[2] Krechowicz M (2020). Comprehensive risk management in horizontal directional drilling projects. J. Constr. Eng. Manag..

[3] Lu H (2020). Trenchless construction technologies for oil and gas pipelines: state-of-the-art review. J. Constr. Eng. Manag..

[4] Sun P, Tian M, Cao H, Niu L, Zhang S (2018). Study on the mechanism of ENI action on preventing drilling fluid overflowing in HDD. Tunn. Undergr. Space Technol..

[5] Bleier R (1990). Selecting a drilling fluid. J. Petrol. Technol..

[6] Mclaren TI, Guppy CN, Tighe MK (2012). A rapid and nondestructive plant nutrient analysis using portable x-ray fluorescence. Soil Sci. Soc. Am. J..

[7] Weindorf DC, Bakr N, Zhu Y (2014). Advances in portable x-ray fluorescence (PXRF) for environmental, pedological, and agronomic applications. Adv. Agron..

[8] Paulette L, Man T, Weindorf DC, Person T (2015). Rapid assessment of soil and contaminant variability via portable x-ray fluorescence spectroscopy: Copşa Mică, Romania. Geoderma.

[9] Qu M, Wang Y, Huang B, Zhao Y (2018). Spatial uncertainty assessment of the environmental risk of soil copper using auxiliary portable X-ray fluorescence spectrometry data and soil pH. Environ. Pollut..

[10] Qu M (2019). Correction of in-situ portable X-ray fluorescence (PXRF) data of soil heavy metal for enhancing spatial prediction. Environ. Pollut..

[11] Vanhoof C, Corthouts VR, Tirez K (2004). Energy-dispersive X-ray fluorescence systems as analytical tool for assessment of contaminated soils. J. Environ. Monit..

[12] Parsons C (2013). Quantification of trace arsenic in soils by field-portable Xray fluorescence spectrometry: considerations for sample preparation and measurement conditions. J. Hazard. Mater..

[13] Weindorf DC, Paulette L, Man T (2013). In-situ assessment of metal contamination via portable X-ray fluorescence spectroscopy: Zlatna, Romania. Environ. Pollut..

[14] Turner A, Poon H, Taylor A, Brown MT (2017). In situ determination of trace elements in Fucus spp. by field-portable-XRF. Sci. Total Environ..

[15] Tavares TR (2019). Simplifying sample preparation for soil fertility analysis by x-ray fluorescence spectrometry. Sensors.

[16] USEPA. Method 6200: Field Portable X-ray Fluorescence Spectrometry for the Determination of Elemental Concentrations in Soil and Sediment (2007). https://www.epa.gov/sites/production/files/2015-12/documents/6200.pdf. Accessed August 6th, 2020.

[17] Weindorf D (2014). Influence of ice on soil elemental characterization via portable x-ray fluorescence spectrometry. Pedosphere.

[18] Daniel J, Penn C, Antonangelo J, Zhang H (2020). Physicochemical characterization of horizontal directional drilling residuals. Sustainability.

[19] Lemiere B, Laperche V, Haouche L, Auger P (2014). Portable XRF and wet materials: application to dredged contaminated sediments from waterways. Geochem. Explor. Environ. Anal..

[20] Hürkamp K, Raab T, Völkel J (2009). Two and three-dimensional quantification of lead contamination in alluvial soils of a historic mining area using field portable X-ray fluorescence (FPXRF) analysis. Geomorphology.

[21] Ge L, Lai W, Lin Y (2004). Influence of and correction for moisture in rocks, soils and sediments on in situ XRF analysis. X-Ray Spectrom..

[22] Kilbride C, Poole J, Hutchings T (2006). A comparison of Cu, Pb, As, Cd, Zn, Fe, Ni and Mn determined by acid extraction/ICP–OES and ex situ field portable X-ray fluorescence analyses. Environ. Pollut..

[23] Hu W, Huang B, Weindorf DC, Chen Y (2014). Metals analysis of agricultural soils via portable x-ray fluorescence spectrometry. Bull. Environ. Contam. Toxicol..

[24] Zhang, H. & Henderson, K. Procedures Used by OSU Soil, Water, and Forage Analytical Laboratory - Oklahoma State University. Procedures Used by OSU Soil, Water, and Forage Analytical Laboratory | Oklahoma State University (2018). https://extension.okstate.edu/fact-sheets/procedures-used-by-osu-soil-water-and-forage-analytical-laboratory.html. Accessed August 11th, 2020

[25] Gavlak, R., Horneck, D., Miller, R.O., and Kotuby-Amacher, J. Soil, plant and water reference methods for the western region. *WCC-103 Publication, Fort Collins, CO* (2003).

[26] Tankersley KB (2017). Geochemical, economic, and ethnographic approaches to the evaluation of soil, salinity, and water management in Chaco Canyon, New Mexico. J. Archaeol. Sci. Rep..

[27] Coronel EG, Bair DA, Brown CT, Terry RE (2014). Utility and limitations of portable x-ray fluorescence and field laboratory conditions on the geochemical analysis of soils and floors at areas of known human activities. Soil Sci..

[28] Mccomb, J. Q., Rogers, C., Han, F. X. & Tchounwou, P. B. Rapid screening of heavy metals and trace elements in environmental samples using portable x-ray fluorescence spectrometer, a comparative study. *Water Air Soil Pollut. 225* (2014). 10.1007/s11270-014-2169-5 PMC4386753 25861136

[29] Shuttleworth, E. L., Evans, M. G., Hutchinson, S. M. & Rothwell, J. J. Assessment of lead contamination in peatlands using field portable XRF. *Water Air Soil Pollut. 225 *(2014).

[30] Weindorf DC, Zhu Y, Chakraborty S, Bakr N, Huang B (2011). Use of portable X-ray fluorescence spectrometry for environmental quality assessment of peri-urban agriculture. Environ. Monit. Assess..

[31] Richards JR (2012). Trace elements in benchmark soils of Oklahoma. Soil Sci. Soc. Am. J..

[32] Towett EK, Shepherd KD, Drake BL (2015). Plant elemental composition and portable X-ray fluorescence (pXRF) spectroscopy: quantification under different analytical parameters. X Ray Spectrom..

[33] Parsons C (2013). Quantification of trace arsenic in soils by field-portable X-ray fluorescence spectrometry: considerations for sample preparation and measurement conditions. J. Hazard. Mater..

[34] Peng JL (2017). Accuracy evaluation of the crop-weather yield predictive models of Italian ryegrass and forage rye using cross-validation. J. Crop Sci. Biotechnol..

[35] Rinaldi M, Losavio N, Flagella Z (2003). Evaluation and application of the OILCROP–SUN model for sunflower in southern Italy. Agric. Syst..

[36] Willmott CJ (1985). Statistics for the evaluation and comparison of models. J. Geophys. Res..

[37] Aboukila EF, Norton JB (2017). Estimation of saturated soil paste salinity from soil-water extracts. Soil Sci..

[38] Kalnicky DJ, Singhvi R (2001). Field portable XRF analysis of environmental samples. J. Hazard. Mater..

[39] Algee B, Callaghan J, Creelman A (1969). Rapid Determination of moisture content in soil samples using high power microwaves. IEEE Trans. Geosci. Electron..

