# Supplementary material for: Development of a rapid field testing method for metals in horizontal directional drilling residuals with XRF sensor
Source: Sci Rep. 2021 Feb 16;11:3901. doi: 10.1038/s41598-021-83584-4 (PMC7887265; doi:10.1038/s41598-021-83584-4)
Supplement: Supplementary file 1 — Supplementary Information [file 41598_2021_83584_MOESM1_ESM.pdf]

Development of a Rapid Field Testing Method for Metals in Horizontal Directional Drilling Residuals with XRF Sensor

Hailin Zhang[1,*], João Antonangelo[1], and Chad Penn[2]

[1]Oklahoma State University, Department of Plant and Soil Sciences, Stillwater, OK 74078, USA
[2]U.S. Department of Agriculture, National Soil Erosion Research, West Lafayette, IN 479072077, USA
[*]hailin.zhang@okstate.edu

Supplementary Table S1. The sum of metal contents in the oven-dried soil after removal of excess water (AREW) and the metal contents in the filtrates (excess water extracts). The delta variation is between the sum of measurements and the initial target concentration (0, 100, 250, and 1000 mg kg^{-1}) to show the recovery of metals spiked. Δ%: delta percentage between the metal contents (sum of oven-dried soil AREW + filtrates) and their initial target concentration (0, 100, 250 and 1,000 mg kg^{-1}).

Soil	------Cr------		------Ni------		------Cu------		------Zn------		------As------		------Cd------		------Pb------	
	mg kg^{-1}	Δ%	mg kg^{-1}	Δ%	mg kg^{-1}	Δ%	mg kg^{-1}	Δ%	mg kg^{-1}	Δ%	mg kg^{-1}	Δ%	mg kg^{-1}	Δ%
	--Portable XRF--													
Perkins	0	-	0	-	0	-	0	-	0	-	0	-	0	-
	158	+58	154	+54	123	+23	132	+32	100	0	100	0	111	+11
	372	+49	313	+25	265	+6	320	+28	221	+12	251	+1	280	+12
	1360	+36	1263	+26	1175	+18	1315	+32	970	-3	1041	+4	1227	+23
LCB	0	-	0	-	0	-	0	-	0	-	0	-	0	-
	91	-9	113	+13	99	-1	100	0	93	-7	83	-17	69	-31
	269	+8	279	+11	238	-5	259	+3	253	+1	224	-11	241	-4
	959	-4	1025	+2	962	-4	1059	+6	962	-4	928	-7	1131	+13
Stillwater	0	-	0	-	0	-	0	-	0	-	0	-	0	-
	68	-32	131	+31	98	-2	105	+5	92	-8	91	-9	84	-16
	235	-6	281	+12	232	-7	277	+11	228	-9	229	-8	265	+6
	1045	+5	1115	+12	1028	+3	1142	+14	972	-3	953	-5	1100	+10
	--ICP (3050B method)--													
Perkins	0	-	0	-	0	-	0	-	0	-	0	-	0	-
	103	+3	124	+24	110	+10	117	+17	103	+3	113	+13	102	+2
	243	-3	286	+15	272	+9	303	+21	246	-2	276	+10	272	+9
	1066	+7	1177	+18	1225	+23	1266	+27	1016	+2	1127	+13	1151	+15
LCB	0	-	0	-	0	-	0	-	0	-	0	-	0	-
	104	+4	107	+7	109	+9	105	+5	98	-2	107	+7	93	-7
	250	0	255	+2	256	+3	250	0	256	+3	250	0	259	+4
	1016	+2	1053	+5	1099	+10	1128	+13	1038	+4	1067	+7	1120	+12
Stillwater	0	-	0	-	0	-	0	-	0	-	0	-	0	-
	101	+1	106	+6	106	+6	106	+6	91	-9	103	+3	102	+2
	268	+7	288	+15	279	+12	290	+16	256	+2	277	+11	288	+15
	1035	+3	1079	+8	1139	+14	1169	+17	1048	+5	1055	+5	1131	+13
